# Supplementary material for: Serum Phospholipids Are Potential Therapeutic Targets of Aqueous Extracts of Roselle (Hibiscus sabdariffa) against Obesity and Insulin Resistance
Source: Int J Environ Res Public Health. 2022 Dec 9;19(24):16538. doi: 10.3390/ijerph192416538 (PMC9779630; doi:10.3390/ijerph192416538)
Supplement: Supplementary file 1 [file ijerph-19-16538-s001.zip › ijerph-2053162-supplementary.pdf]

Supplementary Material

Serum Phospholipids Are Potential Therapeutic Targets of Aqueous Extracts of Roselle (*Hibiscus sabdariffa*) against Obesity and Insulin Resistance

Table S1. Identification parameters of the phospholipids identified in serum samples obtained from rats treated with *Hibiscus sabdariffa* white and purple calyces aqueous extracts.

Compound	Rt [min]	Molecular formula	Expected mass (Da)	Observed mass (Da)	Mass error (ppm)	Adducts	Fragments
Phosphatidic acids (PA)							
LysoPA 16:0	17.98	C19H39O7P	410.2433	409.2341	-4.7	[M-H]-	255.2615
LysoPA 18:1	18.61	C21H41O7P	436.2590	435.2487	-6.8	[M-H]-	281.2167
LysoPA 18:2	16.80	C21H39O7P	434.2433	433.2340	-2.0	[M-H]-	279.2426
LysoPA 20:4	16.75	C23H39O7P	458.2433	457.2342	-4.0	[M-H]-	303.2591
LysoPA 22:6	16.60	C25H39O7P	482.2433	481.2344	-3.5	[M-H]-	327.1625
PA 16:1 18:3	10.22	C37H65O8P	688.4417	667.4351	1.0	[M-H]-	253.1224, 277.2284
PA 22:4 22:6	25.74	C47H73O8P	796.5043	795.4953	-2.2	[M-H]-	327.2225, 331.2933
Phosphatidylcholines (PC)							
LysoPC 16:0	16.72	C24H50NO7P	495.3325	493.3247	-1.0	[M-H]-	255.2248

LysoPC 16:1	15.29	C24H48NO7P	493.3168	492.3078	-3.7	$[M-H]^-$	253.2481
LysoPC 18:0	18.71	C26H54NO7P	523.3638	522.3569	0.7	$[M-H]^-$	283.2794
PC 16:1 16:1	25.65	C40H76NO8P	729.5309	728.5220	-2.1	$[M-H]^-$	253.2471
Phosphatidylethanolamines (PE)							
LysoPE 16:0	15.66	C21H44NO7P	453.2855	452.2765	-4.0	$[M-H]^-$	255.2119
LysoPE 18:0	17.63	C23H48NO7P	481.3168	480.3084	-2.4	$[M-H]^-$	283.1907
LysoPE 18:1	16.12	C23H46NO7P	479.3012	478.2924	-3.2	$[M-H]^-$	281.2429
LysoPE 18:2	14.93	C23H44NO7P	477.2855	476. 2763	-4.1	$[M-H]^-$	279.2370
LysoPE 20:0	17.72	C25H52NO7P	509.3481	508.3409	0.1	$[M-H]^-$	311.2158
LysoPE 20:1	16.19	C25H50NO7P	507.3325	506.3249	-0.6	$[M-H]^-$	309.2644
LysoPE 20:4	14.95	C25H44NO7P	501.2855	500.2770	-2.6	$[M-H]^-$	303.1958
LysoPE 22:6	14.89	C27H44NO7P	525.2855	524.2770	-1.1	$[M-H]^-$	327.2491
PE 16:0 22:4	24.88	C43H78NO8P	767.5465	767.5448	-2.2	$[M-H]^-$	255.1984, 331.2012
PE 18:0 20:3	22.59	C43H80NO8P	769.5622	768.5509	-5.2	$[M-H]^-$	283.2428, 305.2435
PE 18:0 20:5	24.19	C43H76NO8P	765.5309	764.5215	-2.9	$[M-H]^-$	283.2117, 301.2443
PE 18:1 18:2	24.82	C41H76NO8P	741.5309	740.5214	-3.0	$[M-H]^-$	279.2302, 281.2594
PE 18:1 22:5	24.40	C45H78NO8P	791.5465	790.5396	0.4	$[M-H]^-$	281.2588, 327.2320
PE 20:2 22:6	23.94	C47H78NO8P	815.5465	814.5357	-4.3	$[M-H]^-$	307.2326, 327.2375
Phosphatidylinositol (PI)							
LysoPI 16:0	16.12	C25H49O12P	572.2962	571.2871	-1.8	$[M-H]^-$	255.2351
LysoPI 18:0	19.26	C27H53O12P	600.3275	599.3159	-7.2	$[M-H]^-$	283.2368
LysoPI 20:4	15.29	C29H49O12P	620.2962	619.2832	-9.2	$[M-H]^-$	303.2860
PI 18:1 18:1	25.87	C45H83O13P	862.5571	861.5465	-3.9	$[M-H]^-$	281.2801
PI 16:0 22:6	23.55	C47H79O13P	882.5258	881.5194	1.0	$[M-H]^-$	255.2404, 327.3281
PI 16:1 20:3	26.23	C45H79O13P	858.5258	857.5189	0.4	$[M-H]^-$	253.2543, 305.2212
PI 18:0 20:5	25.52	C47H81O13P	884.5415	883.5306	-4.1	$[M-H]^-$	283.25836, 301.2404
PI 20:1 22:4	15.22	C51H89O13P	940.6041	939.5960	-0.9	$[M-H]^-$	301.2415, 331.1925
Phosphatidylserine (PS)							
LysoPS 16:0	15.89	C22H44NO9P	497.2754	496.2679	-0.5	$[M-H]^-$	255.2308
LysoPS 16:1	14.50	C22H42NO9P	495.2597	494.2515	-2.0	$[M-H]^-$	253.2225
LysoPS 18:0	18.28	C24H48NO9P	525.3067	524.2996	0.3	$[M-H]^-$	283.2331
LysoPS 18:1	16.43	C24H46NO9P	523.2910	522.2834	-0.7	$[M-H]^-$	281.2136
LysoPS 18:2	15.10	C24H44NO9P	521.2754	520.2670	-2.0	$[M-H]^-$	279.2417

LysoPS 20:5	14.12	C26H42NO9P	543.2597	542.2512	-2.4	$[M-H]^-$	301.2112
PS 16:0 22:2	15.80	C44H82NO10P	815.5676	814.5594	-1.2	$[M-H]^-$	255.2272, 335.1519
PS 16:0 22:6	15.69	C44H74NO10P	807.5050	806.4994	2.0	$[M-H]^-$	255.2732, 327.2681
PS 18:0 18:1	25.66	C42H80NO10P	789.5520	788.5444	-0.4	$[M-H]^-$	281.2228, 283.2636
PS 18:0 22:6	17.73	C46H78NO10P	835.5363	834.5330	4.7	$[M-H]^-$	283.2622, 327.1738
PS 20:0 20:3	15.84	C46H84NO10P	841.5833	840.5737	-2.7	$[M-H]^-$	305.2082, 311.2229
PS 20:0 22:6	17.76	C48H82NO10P	863.5676	862.5618	1.6	$[M-H]^-$	311.2318, 327.2270
